# In Memoriam

**Published:** 1887-06

**Authors:** 


### IN MEMORIAM—HENRY DETWILER, M. D.

In the death of Dr. Detwiler Homœopathy loses its oldest practitioner, and one who has ably upheld Homœopathy for over half a century. The places of such men can hardly be filled. One by one the old veterans drop off and, alas! none seem to arise to continue their work.

Dr. Henry Detwiler died at his home, in Easton, Pa., April 21st. He was the oldest citizen of Easton, the man who dispensed the first homœopathic remedy in America, and believed to be the oldest homœopathic physician in the world. He was born in Switzerland December 18th, 1795, and consequently was in his ninety-second year. He landed in Philadelphia in July, 1817, practiced there a short time, moved to Allentown, and then to Hellertown. Dr. Detwiler gained great prominence all through the section in 1818 by checking an epidemic which was carrying off hundreds of people. The cause was learned by him to be poison in the lining of earthern jars then in common use. It made him popular from that time on, and his medical practice has been enormous. He was a personal friend of Hahnemann and has been repeatedly honored in homœopathic circles. His progenity is great, six children, twenty-seven grandchildren, twenty-one great-grandchildren, and two great-great-grandchildren surviving him. His sons are Dr. J. J. Detwiler, Easton, and Dr. W. F. Detwiler, Hellertown, both prominent men.

Dr. Detwiler was an able man; was remarkable for his fund of knowledge on natural sciences. He had lost none of his old-time interest in public affairs because of his many years, and during the past few years attended nearly every performance at the Able Opera House. His mind to the last was as unclouded as that of a boy, due in a great measure to the excellent care he had taken of himself.

